# RNA-Seq analysis and *de novo* transcriptome assembly of Cry toxin susceptible and tolerant *Achaea janata* larvae

**DOI:** 10.1038/s41597-019-0160-0

**Published:** 2019-08-22

**Authors:** Narender K. Dhania, Vinod K. Chauhan, R. K. Chaitanya, Aparna Dutta-Gupta

**Affiliations:** 1 Department of Animal Biology, School of Life Sciences, University of Hyderabad, Hyderabad, 500046 India; 2 Department of Animal Sciences, School of Basic and Applied Sciences, Central University of Punjab, Bathinda, 151001 India

**Keywords:** Transcriptomics, Entomology, RNA sequencing

## Abstract

Larvae of most lepidopteran insect species are known to be voracious feeders and important agricultural pests throughout the world. *Achaea janata* larvae cause serious damage to *Ricinus communis* (Castor) in India resulting in significant economic losses. Microbial insecticides based on crystalline (Cry) toxins of *Bacillus thuringiensis* (Bt) have been effective against the pest. Excessive and indiscriminate use of Bt*-*based biopesticides could be counter-productive and allow susceptible larvae to eventually develop resistance. Further, lack of adequate genome and transcriptome information for the pest limit our ability to determine the molecular mechanisms of altered physiological responses in Bt-exposed susceptible and tolerant insect strains. In order to facilitate biological, biochemical and molecular research of the pest species that would enable more efficient biocontrol, we report the midgut *de novo* transcriptome assembly and clustering of susceptible Cry toxin-exposed and Cry toxin tolerant *Achaea janata* larvae with appropriate age-matched and starvation controls.

## Abstract (summary)

**Table:** 

Design Type(s)	parallel group design • transcription profiling by high throughput sequencing design
Measurement Type(s)	transcription profiling assay
Technology Type(s)	RNA sequencing
Factor Type(s)	toxin disposition • toxin exposure • temporal_interval • technical replicate
Sample Characteristic(s)	Achaea janata • midgut

Machine-accessible metadata file describing the reported data (ISA-Tab format)

## Background & Summary

*Bacillus thuringiensis* (Bt) insecticidal proteins used in sprayable formulations and transgenic crops are the most promising alternatives to synthetic insecticides. However, the evolution of resistance in the field, as well as laboratory insect populations is a serious roadblock to this technology. *Achaea janata*, a major castor crop pest in India, is controlled using Bt-based formulation^1^ comprising of *Cry1* (*Cry1Aa*, *Cry1Ab*, and *Cry1Ac*) and *Cry2* (*Cry2Aa* and *Cry2Ab*) genes^2^. Recent studies from our group reported extensive changes at the cellular and molecular level in the midgut of *A*. *janata* exposed to a sublethal dose of the *Bt* formulation^3–5^. Since a decade, reports of resistance against Bt toxins and their mechanisms have been emerging^6,7^. Long term exposure to Cry toxin formulations promotes tolerance in larvae which eventually leads to resistance^6–8^. Development of Bt resistance could be due to alterations in proteolytic cleavage of the Cry toxin, altered receptor binding or enhanced midgut regeneration responses^9–11^. With the advent of next generation sequencing technology it is now possible to characterize the entire repertoire of transcripts under different conditions and predict pathways involved in various molecular mechanisms. The RNA sequencing study presented here generated the first *de novo* transcriptome assembly of castor semilooper, *Achaea janata* (Noctuidae: Lepidoptera), and compared gene expression signatures between toxin-exposed susceptible and tolerant larvae. This article, is a first step in determining the primary basis for Cry tolerance in the pest, which could facilitate new long term management strategies.

## Methods

### Toxin administration and sample preparation

**Fig. 1:**
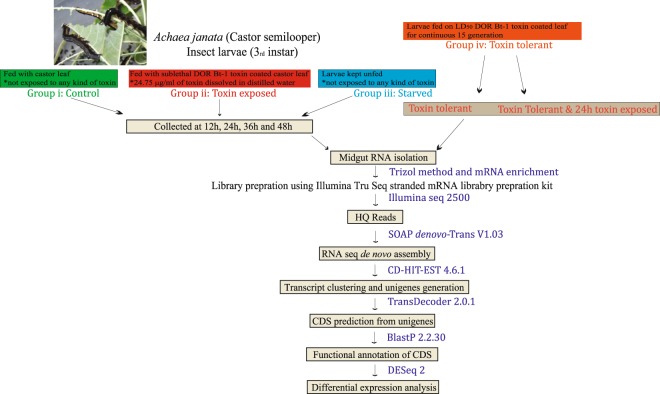
Flow chart showing the methodology used for the present study.

Wild population of *A*. *janata* larvae, unexposed to pesticides, was field collected from the Indian Institute of Oil Seed Research, Hyderabad, India. Further, the larvae were reared and maintained on castor leaves at 27 ± 2 °C, 14:10 h (light: dark) photoperiod and 60–70% relative humidity for three generations at the insectary of School of Life Sciences, University of Hyderabad, India. In the present *de novo* transcriptome analysis for the sublethal toxin exposure, 1/10 of LD_50_ was used (Group ii) (Fig. 1), while for the generation of a tolerant population (Group iv) (Fig. 1) an LD_50_ dose of DOR Bt-1 formulation was administered^1^. Larval batches (n = 100) designated as Cry-susceptible larvae and control larvae were exposed to toxin-coated and distilled water coated leaves respectively. The midgut was isolated from 15 randomly selected surviving larvae from each batch after every 12 h till 48 h. In earlier study we noticed that larvae probably sense the toxin and avoid feeding on toxin coated leaves after a short exposure. Hence, to eliminate any effect induced by starvation, an additional batch (Group iv) of 3^rd^ instar larvae was maintained on moist filter paper and collected for the midgut isolation every 12 h till 48 h. All the midgut dissections were carried out in ice-cold insect Ringer solution (130 mM NaCl, 0.5 mM KCl, and 0.1 mM CaCl_2_). The experiment was performed in duplicates. For the Cry tolerant larval population, larvae (n = 100) in each generation were exposed to LD_50_ dose and the surviving insects were maintained for larval development, pupal molting, adult emergence and egg laying. The larvae hatched from the eggs were collected and reared till 3^rd^ larval instar larvae and exposed to LD_50_ Bt dose once again. This schedule was carried out for fifteen generations. The batch (n = 100) of Cry tolerant larvae thus generated were exposed to toxin-coated leaves and the midguts were isolated from randomly selected fifteen larvae after 24 h. Total RNA was isolated from the midgut samples using Trizol-based method. The RNA was quantified using NanoDrop^TM^ 8000 spectrophotometer and the quality was assessed using 1% formaldehyde denaturing agarose gel.

### Library preparation

Illumina 2 × 150 pair end libraries were prepared as follows. Briefly, mRNA was enriched from isolated total RNA and fragmented. The fragmented mRNA was used for first-strand cDNA synthesis, followed by second-strand generation, A-tailing and adapter ligation. Adapter ligated products were purified and PCR amplification was carried out. PCR amplified cDNA libraries were assessed for quality and quantity using DNA High Sensitivity Assay Kit (Agilent Technologies).

### Quality assessment prior to cluster generation and sequencing

The amplified libraries were analyzed using Bioanalyzer 2100 and High Sensitivity DNA chip (Agilent Technologies). After obtaining the Qubit concentration for each of the libraries, it was loaded on Illumina platform (2 × 150 bp chemistry) for cluster generation and sequencing. Data was generated on Illumina HiSeq. 2500 system and paired-end sequencing allowed the template fragments to be sequenced in both the forward and reverse directions. The library molecules bind to complementary adapter oligos on paired-end flow cell. The adapters were designed to allow selective cleavage of the forward strands after re-synthesis of the reverse strand during sequencing. The copied reverse strand was then used to sequence from the opposite end of the fragment. Total RNA was subjected to pair-end library preparation with Illumina TruSeq Stranded Total RNA Library Preparation Kit. The mean size of the libraries was between 357 bp to 567 bp for the 28 samples. The libraries were sequenced and high quality data was generated for ~ 3.05 GB data per sample (Online-only Table 1).

### Sequence analysis

Illumina 2 × 150 pair end libraries were prepared using the Illumina TruSeq stranded mRNA Library Preparation Kit and as per the firm’s protocol (Illumina Inc.). The amplified libraries were analyzed on the Bioanalyzer 2100 with a High Sensitivity DNA chip (Agilent Technologies). The *de novo* master assembly was generated using “SOAP-denovo-Trans (v1.03)” assembler (Short Oligonucleotide Analysis Package)^12^. For each data set, raw quality was assessed and filtered with Trimmomatic (v.0.36)^13^. Transcripts were clustered using the CD-HIT (Cluster Database at High Identity with Tolerance) package^14^. The predicted proteins of CDS (Coding sequence) were subjected to similarity search against NCBI’s non-redundant (nr) database using the BLASTP (Basic Local Alignment Search Tool) algorithm.

## Data Records

The total raw sequencing data from 28 samples (14 biological replicates, where the sequencing experiment was performed twice and the replicates are derived from different pool of larvae and they are biologically independent samples) was used for assembly in the present study. They have been deposited in the NCBI SRA database, with identifier SRP18670^15^ and accession numbers SRR8617834, SRR8617835, SRR8617836, SRR8617837, SRR8617838, SRR8617839, SRR8617840, SRR8617841, SRR8617842, SRR8617843, SRR8617844, SRR8617845, SRR8617846, SRR8617847, SRR8617848, SRR8617849, SRR8617850, SRR8617851, SRR8617852, SRR8617853, SRR8617854, SRR8617855, SRR8617856, SRR8617857, SRR8617858, SRR8617859, SRR8617860 and SRR8617861, under BioProject PRJNA523326 and BioSample SAMN09241884. This Transcriptome Shotgun Assembly project has been deposited at DDBJ/ENA/GenBank under the accession GHGZ00000000^16^. The version described in this paper is the first version, GHGZ01000000.

## Technical Validation

SOAPdenovo-Trans assembler was used to generate *de novo* transcriptome assembly from four experimental sets of midgut samples viz. Group (i) susceptible larvae exposed to medium (water), Group (ii) susceptible larvae exposed to 1/10 of LD_50_ dosage of DOR Bt-1 formulation, Group (iii) susceptible larvae subjected to starvation and Group (iv) tolerant larvae exposed to LD_50_ dosage of DOR Bt-1 formulation (reared for 15 generations) (Fig. 1). A total of 1,74,066 transcripts were generated for master assembly with a transcriptome length of 10,02,47,510 bps (base pairs). A total of 1,36,818 unigenes were reported using CD-HIT and 35,559 coding sequences were predicted by Transdecoder. The top-hit species distribution revealed that majority (23%) of the CDS aligned with *Spodoptera litura* followed by *Helicoverpa armigera* and *Heliothis virescens* all of which belong to family Noctuidae in the Lepidoptera order.

### Transcriptome assembly

The *de novo* master assembly of high quality reads of 28 processed samples was accomplished using “SOAP-denovo-Trans (v1.03)” assembler^12^. For each data set, raw quality (phred40) was assessed and filtered with Trimmomatic (v.0.36) using the parameters ILLUMINACLIP:adapter.fasta:2:30:8 MINLEN:40 to remove adaptor sequence and filter by quality score^13^. An average of 19 million clean reads were obtained. Statistics of high quality reads with total reads, base count and data size are summarized in Online-only Table 1 and statistics of assembled transcripts as well as length distribution is presented in Table 1.

**Table 1:** Statistics of assembled transcripts and transcript length distribution.

Description	Master Assembly
Total number of transcripts	1,74,066
Total transcriptome length in bps	100,247,510
Average transcript length in bps	575
N50	421
Maximum transcript length in bps	25,338
Minimum transcript length in bps	200
**Metrics**	**Master Assembly**
Length >=200 & <=300	85429
Length >300 & <=400	32056
Length >400 & <=500	13439
Length >500 & <=600	8124
Length >600 & <=700	5796
Length >700 & <=800	4098
Length >800 & <=900	3028
Length >900 & <=1000	2444
Length >1000 & <=5000	18383
Length >5000	1269

### Clustering

To filter the redundancy or the noise, it was required to select one representative transcript for transcripts clusters. Transcripts were clustered using CD-HIT (Cluster Database at High Identity with Tolerance) package^14^. CD-HIT-EST v4.6.1 was used to remove the shorter redundant transcripts when they were 100% covered by other transcripts with more than 90% identity. The non-redundant clustered transcripts were then designated as unigenes (Table 2). CDS were predicted from the unigene sequences with Transdecoder at default parameters which resulted in the identification of 35,559 CDS (Table 3).

**Table 2:** Statistics of unigenes and length distribution.

Description	Unigenes
Total number of unigenes	1,36,618
Total size of all unigenes in bps	86,577,226
Average unigene length in bps	633
N50	458
Maximum unigene length in bps	25,338
Minimum unigene length in bps	200
**Metrics**	**Unigenes**
Length >=200 & <=300	56403
Length >300 & <=400	26874
Length >400 & <=500	12354
Length >500 & <=600	7740
Length >600 & <=700	5647
Length >700 & <=800	3980
Length >800 & <=900	2941
Length >900 & <=1000	2366
Length >1000 & <=5000	17193
Length >5000	1120

**Table 3:** Statistics and length distribution of the predicted CDS.

Description	Metrics
Total number of cds	35,559
Total size of all cds in bps	25,527,927
Average cds length in bps	717
Maximum cds length in bps	8,595
**Metrics**	**CDS**
Length >200 & <=300	761
Length >300 & <=400	9515
Length >400 & <=500	5643
Length >500 & <=600	4127
Length >600 & <=700	3003
Length >700 & <=800	2275
Length >800 & <=900	1867
Length >900 & <=1000	1652
Length >1000 & <=5000	6684
Length >5000	32

### Annotation

**Fig. 2:**
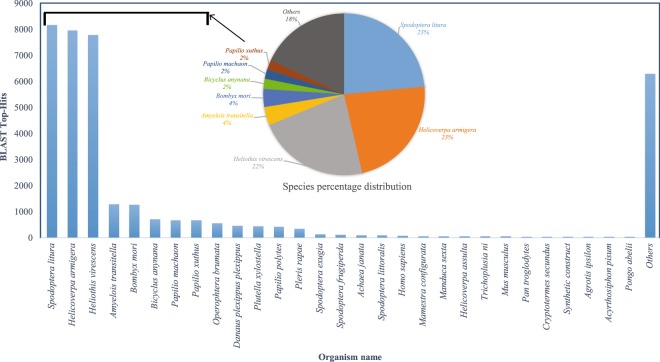
Top-hit species distribution of most closely related insect species demonstrated using a horizontal bar graph.

**Fig. 3:**
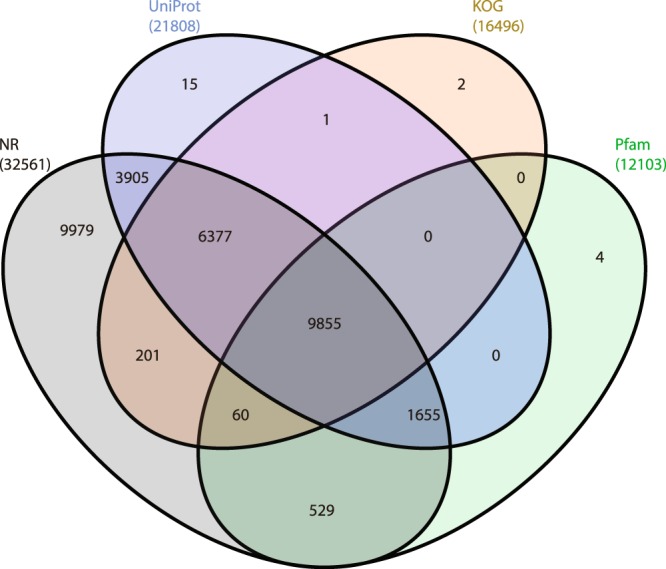
Venn diagram representation of annotated protein in different databases.

The predicted proteins of CDS were subjected to similarity search against NCBI’s non-redundant (nr) database using the BLASTP algorithm. Out of total 35,559 proteins, 32,561 proteins were captured with hits and 2,998 with no hits (Annotation of each transcript of the assembled transcriptome)^17^. The top-hit species distribution revealed that majority of the hits were found to be against the species *Spodoptera litura* followed by *Helicoverpa armigera* and *Heliothis virescens* (Fig. 2). Simultaneously, all protein sequences were searched for similarity against NR, UniProt (Universal Protein Resources), KOG (EuKaryotic Orthologous Groups) and Pfam database using BLASTP with an e-value threshold of 1e^−5^. The BLAST result of four databases has resulted in Fig. 3.

### Differential expression

In this work we compared the control and Cry toxin tolerant larval transcript map reads for the differential expression analysis. Analysis of count data was done using DESeq. 2 in RStudio platform^18^. Differential expression analysis shows significant differences in the tolerant larval population as compared to the susceptible population (Differential expression analysis)^17^. Out of 35,559 CDS analysed, 320 CDS show significant variation (padj < 0.05). Few of these genes like (i) gi|1131919362| Ca^2+^-binding protein, RTX toxin-related, (ii) gi|1199381583| superoxide dismutase [Cu-Zn] 2-like, (iii) gi|315139350| serine protease 63, (iv) gi|1274141826| trypsin, alkaline C-like and (v) gi|1274136486| apolipophorins isoform X2 were shown to be upregulated, while (i) gi|123995301| ribosomal protein SA, (ii) gi|744619941| predicted: 60 S ribosomal protein L8, (iii) gi|45219787| ribosomal protein S3A, (iv) gi|1344818460| alanine aminotransferase 1-like and (v) gi|501300966| ubiquitin were downregulated.

### ISA-Tab metadata file

Download metadata file

## Data Availability

The following software version/script were used in the current manuscript. The RStudio software packages are available open-source from the repository at https://www.rstudio.com/. SOAPdenovo-Trans (v.1.03)^12^. Trimmomatic (v.0.36)^13^. CD-HIT-EST (v.4.6.1)^14^. BlastP (v.2.2.30). The DESeq. 2 scripts were used for plotting the differential expression data. https://bioconductor.org/packages/release/bioc/vignettes/DESeq. 2/inst/doc/DESeq. 2.html. As an input we have used- (1) a table having RAW read counts and (2) metadata, that is, each line contains description of one of the samples. See example below: #SampleName Condition. C1_raw_read_count control. D1_raw_read_count tolerant.

## Online-only Table

**Online-only Table 1:** Statistics of high quality data obtained from midgut samples of *A*. *janata* larva.

Sample Id	Reads	Total reads	Bases	Total bases	Data in Gb
Contorl_12h_R1.fq	10028942	20057884	1.494E + 09	2.989E + 09	3
Contorl_12h_R2.fq	10028942		1.494E + 09		
Contorl_12h_replicate_R1.fq	8628916	17257832	1.291E + 09	2.582E + 09	2.6
Contorl_12h_replicate_R2.fq	8628916		1.291E + 09		
Contorl_24h_R1.fq	8034841	16069682	1.201E + 09	2.401E + 09	2.4
Contorl_24h_R2.fq	8034841		1.201E + 09		
Contorl_24h_replicate_R1.fq	6983555	13967110	1.041E + 09	2.082E + 09	2.1
Contorl_24h_replicate_R2.fq	6983555		1.041E + 09		
Contorl_36h_R1.fq	8192427	16384854	1.226E + 09	2.452E + 09	2.5
Contorl_36h_R2.fq	8192427		1.226E + 09		
Contorl_36h_replicate_R1.fq	15482277	30964554	2.301E + 09	4.601E + 09	4.6
Contorl_36h_replicate_R2.fq	15482277		2.301E + 09		
Contorl_48h_R1.fq	8122611	16245222	1.215E + 09	2.431E + 09	2.4
Contorl_48h_R2.fq	8122611		1.215E + 09		
Contorl_48h_replicate_R1.fq	9181499	18362998	1.368E + 09	2.736E + 09	2.7
Contorl_48h_replicate_R2.fq	9181499		1.368E + 09		
Starved_12h_R1.fq	9799368	19598736	1.436E + 09	2.873E + 09	2.9
Starved_12h_R2.fq	9799368		1.436E + 09		
Starved_12h_replicate_R1.fq	11031631	22063262	1.645E + 09	3.29E + 09	3.3
Starved_12h_replicate_R2.fq	11031631		1.645E + 09		
Starved_24h_R1.fq	7656143	15312286	1.147E + 09	2.294E + 09	2.3
Starved_24h_R2.fq	7656143		1.147E + 09		
Starved_24h_replicate_R1.fq	10655240	21310480	1.59E + 09	3.181E + 09	3.2
Starved_24h_replicate_R2.fq	10655240		1.59E + 09		
Starved_36h_R1.fq	9079024	18158048	1.355E + 09	2.711E + 09	2.7
Starved_36h_R2.fq	9079024		1.355E + 09		
Starved_36h_replicate_R1.fq	9110626	18221252	1.357E + 09	2.714E + 09	2.7
Starved_36h_replicate_R2.fq	9110626		1.357E + 09		
Starved_48h_R1.fq	10697833	21395666	1.598E + 09	3.196E + 09	3.2
Starved_48h_R2.fq	10697833		1.598E + 09		
Starved_48h_replicate_R1.fq	7723461	15446922	1.149E + 09	2.297E + 09	2.3
Starved_48h_replicate_R2.fq	7723461		1.148E + 09		
Toxin_exposed_12h_R1.fq	16730802	33461604	2.493E + 09	4.986E + 09	5
Toxin_exposed_12h_R2.fq	16730802		2.493E + 09		
Toxin_exposed_12h_replicate_R1.fq	10576431	21152862	1.579E + 09	3.158E + 09	3.1
Toxin_exposed_12h_replicate_R2.fq	10576431		1.579E + 09		
Toxin_exposed_24h_R1.fq	10730932	21461864	1.594E + 09	3.188E + 09	3.2
Toxin_exposed_24h_R2.fq	10730932		1.594E + 09		
Toxin_exposed_24h_replicate_R1.fq	10807754	21615508	1.612E + 09	3.225E + 09	3.2
Toxin_exposed_24h_replicate_R2.fq	10807754		1.612E + 09		
Toxin_exposed_36h_R1.fq	10061786	20123572	1.5E + 09	3E + 09	3
Toxin_exposed_36h_R2.fq	10061786		1.5E + 09		
Toxin_exposed_36h_replicate_R1.fq	10027587	20055174	1.49E + 09	2.98E + 09	3
Toxin_exposed_36h_replicate_R2.fq	10027587		1.49E + 09		
Toxin_exposed_48h_R1.fq	9080458	18160916	1.332E + 09	2.664E + 09	2.7
Toxin_exposed_48h_R2.fq	9080458		1.332E + 09		
Toxin_exposed_48h_replicate_R1.fq	6058585	12117170	900542510	1.801E + 09	1.8
Toxin_exposed_48h_replicate_R2.fq	6058585		900375353		
Toxin_tolerant_R1.fq	10917204	21834408	1.617E + 09	3.234E + 09	3.2
Toxin_tolerant_R2.fq	10917204		1.617E + 09		
Toxin_tolerant_replicate_R1.fq	14952551	29905102	2.225E + 09	4.451E + 09	4.5
Toxin_tolerant_replicate_R2.fq	14952551		2.225E + 09		
Toxin_tolerant_24h_exposed_R1.fq	7372799	14745598	1.094E + 09	2.189E + 09	2.2
Toxin_tolerant_24h_exposed_R2.fq	7372799		1.094E + 09		
Toxin_tolerant_24h_exposed_replicate_R1.fq	18861211	37722422	2.814E + 09	5.627E + 09	5.6
Toxin_tolerant_24h_exposed_replicate_R2.fq	18861211		2.814E + 09		
